# In vivo administration of urolithin A and B prevents the occurrence of cardiac dysfunction in streptozotocin-induced diabetic rats

**DOI:** 10.1186/s12933-017-0561-3

**Published:** 2017-07-06

**Authors:** Monia Savi, Leonardo Bocchi, Pedro Mena, Margherita Dall’Asta, Alan Crozier, Furio Brighenti, Donatella Stilli, Daniele Del Rio

**Affiliations:** 1 Department of Food and Drugs, University of Parma, Parco Area delle Scienze 27/A, 43124 Parma, Italy; 2 Department of Chemistry, Life Sciences and Environmental Sustainability, University of Parma, Parco Area delle Scienze 11/A, 43124 Parma, Italy; 3 Department of Nutrition, University of California, 3143 Meyer Hall One Shields Avenue, Davis, CA 95616-5270 USA

**Keywords:** Diabetes, Ellagitannins, Cardiac performance, Cardiomyocyte mechanics, Urolithins

## Abstract

**Background:**

Emerging evidence suggests that specific (poly)phenols may constitute new preventative strategies to counteract cell oxidative stress and myocardial tissue inflammation, which have a key role in the patho-physiology of diabetic cardiomyopathy. In a rat model of early diabetes, we evaluated whether in vivo administration of urolithin A (UA) or urolithin B (UB), the main gut microbiota phenolic metabolites of ellagitannin-rich foods, can reduce diabetes-induced microenvironmental changes in myocardial tissue, preventing cardiac functional impairment.

**Methods:**

Adult Wistar rats with streptozotocin-induced type-1 diabetes (n = 29) were studied in comparison with 10 control animals. Diabetic rats were either untreated (n  =  9) or subjected to daily i.p. injection of UA (n = 10) or UB (n = 10). After 3 weeks of hyperglycaemia, hemodynamics, cardiomyocyte contractile properties and calcium transients were measured to assess cardiac performance. The myocardial expression of the pro-inflammatory cytokine fractalkine and proteins involved in calcium dynamics (sarcoplasmic reticulum calcium ATPase, phospholamban and phosphorylated phospholamban) were evaluated by immunoblotting. Plasma, urine and tissue distribution of UA, UB and their phase II metabolites were determined.

**Results:**

In vivo urolithin treatment reduced by approximately 30% the myocardial expression of the pro-inflammatory cytokine fractalkine, preventing the early inflammatory response of cardiac cells to hyperglycaemia. The improvement in myocardial microenvironment had a functional counterpart, as documented by the increase in the maximal rate of ventricular pressure rise compared to diabetic group (+18% and +31% in UA and UB treated rats, respectively), and the parallel reduction in the isovolumic contraction time (−12%). In line with hemodynamic data, both urolithins induced a recovery of cardiomyocyte contractility and calcium dynamics, leading to a higher re-lengthening rate (+21%, on average), lower re-lengthening times (−56%), and a more efficient cytosolic calcium clearing (−32% in tau values). UB treatment also increased the velocity of shortening (+27%). Urolithin metabolites accumulated in the myocardium, with a higher concentration of UB and UB-sulphate, potentially explaining the slightly higher efficacy of UB administration.

**Conclusions:**

In vivo urolithin administration may be able to prevent the initial inflammatory response of myocardial tissue to hyperglycaemia and the negative impact of the altered diabetic milieu on cardiac performance.

## Background

Diabetic cardiomyopathy (DCM), defined as ventricular dysfunction in the absence of concurrent coronary artery disease and/or hypertension, constitutes one of the major complication of both type-1 and type-2 diabetes [1]. Since the early stages of diabetes, the altered tissue microenvironment associated with metabolic changes, including hyperglycaemia, cell oxidative stress and up-regulation of inflammatory cytokines, can directly affect multiple intracellular molecular pathways which impair cardiomyocyte contractility and promote structural damage [1–4].

In the last decade, several studies have focused on novel therapeutic adjuvant strategies to counteract early hyperglycaemia-induced changes in myocardial microenvironment and the associated morpho-functional alterations in the diabetic myocardial tissue [5–7]. In this context, specific attention has recently been devoted to different dietary (poly)phenols which have been shown to be potentially bioactive due to their antioxidant and anti-inflammatory properties [2, 8], leading to cardioprotection, in both diabetic and ischemic hearts. Among these compounds, ellagitannins (ETs), a subclass of hydrolysable tannins present mainly in raspberries, strawberries, pomegranate, walnuts, and oak-aged red wines [9], are known to exert different bioactivities [10, 11]. ETs are hydrolysed in the gut to release ellagic acid, which is further processed by the gut microbiota into a series of metabolites named urolithins, each characterised by a specific hydroxylation pattern. Urolithin A (UA, 3,8-dihydroxy-urolithin) and urolithin B (UB, 3-hydroxy-urolithin) are the main urolithins detected in human biological fluids and tissues after ET consumption by humans, while other urolithins such as urolithin C (UC, 3,8,9-trihydroxy-urolithin) and urolithin D (UD, 3,4,8,9-tetrahydroxy-urolithin) may be found at lower concentrations [9, 10, 12]. Urolithins can be further metabolised, mainly by enterocytes and hepatocytes, by phase II enzymes yielding glucuronide, sulphate, and methyl derivatives which appear in the circulation at high nM to low µM concentrations. It has been shown that UB, UB-glucuronide and UD, at a concentration coherent with a dietary exposure to ET-rich foods, are able to modulate the pro-inflammatory mediators secreted by cultured neonatal cardiac cells, cardiomyocytes and fibroblasts, after 3-weeks exposure to high glucose concentrations [13]. Specifically, UB and UB-glucuronide prevented inflammatory responses in cardiomyocytes, while UD reduced the over-expression of the pro-inflammatory cytokine fractalkine by cardiac fibroblasts. However, no in vivo data are currently available demonstrating the functional counterpart of the positive impact of urolithins on diabetes-induced microenvironmental changes.

This paper specifically addresses this issue in order to investigate the effects of urolithin administration on the recovery of cardiac function, as measured at organ and cellular levels, and on the inflammatory state of myocardial tissue in a rat model of early diabetes induced by streptozotocin. The presence of urolithin metabolites in the cardiac tissue and other biological tissues/fluids was also assessed to better understand the effect of these phenolic metabolites on DCM prevention.

## Methods

The investigation was approved by the Veterinary Animal Care and Use Committee of the University of Parma-Italy (Prot. No. 59/12) and conforms to the National Ethical Guidelines of the Italian Ministry of Health and the Guide for the Care and Use of Laboratory Animals (National Institute of Health, Bethesda, MD, USA, revised 1996). All experiments were carried out in accordance with the approved guidelines.

### Animals and experimental protocol

The study population consisted of 39 male Wistar rats (*Rattus norvegicus*) aged 12–14 weeks, weighing 376.4 ± 4.9 g (standard error of the mean, SEM). Animals were kept in unisexual groups of four individuals from weaning, 4 weeks after birth, until the onset of the experiments, in a temperature-controlled room at 22–24 °C, illuminated from 0700 to 1900 h. The bedding of the cages consisted of wood shavings, and food and water were freely available. Type-1 diabetes was induced in 29 animals (group D) by a single intra-peritoneal (i.p.) injection of streptozotocin (STZ, 60 mg/kg, Sigma-Aldrich, Milan, Italy) while the remaining 10 control rats (CTRL group) were injected only with the vehicle (0.9% NaCl). Glucose blood levels and body weights were measured in 4-h-fasting animals, before STZ or vehicle injection, then after 2 days, and weekly until sacrifice 3 weeks after the induction of hyperglycaemia. D animals were either untreated (D3 group, n  =  9) or subjected to daily i.p. injection of UA (Toronto Research Chemicals Inc., Toronto, ON, Canada) (D3-UA, n = 10) or UB (Toronto Research Chemicals) (D3-UB, n = 10), at a dose of 2.5 mg/kg/day.

The treatment started immediately after the documented increase in blood glucose levels (2 days after STZ injection; blood glucose cut-off: 250 mg/dl). To prepare UA and UB stock solutions, the compounds were dissolved in dimethyl sulfoxide (DMSO, Sigma-Aldrich) at 50 mg/ml and stored in darkness at 4 °C. For each animal, immediately prior to i.p. injection, an appropriate aliquot was taken from the stock solution and diluted in PBS to reach the desired concentration in a final volume of 250 µl.

Hemodynamic measurements were performed under anaesthesia in all CTRL rats and in all D animals after 3 weeks of hyperglycaemia (D3, D3-UA and D3-UB). This early stage of diabetes is characterised by the occurrence of the first signs of cardiomyocyte contractile dysfunction associated with changes in myocardial tissue microenvironment, in the absence of marked cardiac structural damage, collagen accumulation or inflammatory cell infiltrate [2].

Immediately after hemodynamic measurements, in subgroups of each experimental group, the heart was excised and cardiac cells were enzymatically isolated to measure cardiomyocyte mechanical properties and calcium transients. From the remaining rats, blood and urine samples were collected by inferior vena cava withdrawal and direct puncture of the exposed bladder, respectively. Urine samples were stored at −20 °C, while blood was centrifuged (2680*g* for 15 min at 4 °C) to obtain plasma. Then, after pancreas and liver removal, the heart was rapidly excised and perfused with a 0.9% NaCl solution to drain the residual blood. Finally, the tissues (left and right ventricles, liver, and pancreas) were wiped with filter paper, weighted, mechanically fragmented in liquid nitrogen and stored at −80 °C. Plasma, urine and tissues were used for determining the distribution of UA, UB and their metabolites. Heart tissue was also used for molecular analyses.

### Plasma, urine and tissue processing prior to UHPLC-MS/MS analysis

Plasma samples were defrosted, vortexed, and extracted as previously reported [14]. Briefly, 400 μl plasma aliquots were mixed with 1 ml of acidified acetonitrile (2% formic acid, Sigma-Aldrich). Samples were vortexed, ultrasonicated for 10 min, and centrifuged at 16,000*g* for 10 min. The supernatant was reduced to dryness using a Speedvac concentrator (Thermo Fisher Scientific Inc., San José, CA, USA), the pellet was then suspended in 100 μl of 50% (v:v) methanol (Sigma-Aldrich) acidified with 0.1% (v:v) formic acid, and centrifuged at 16,000*g* for 5 min prior to analysis by ultra-high performance liquid chromatography-mass spectrometry (UHPLC-MS/MS).

Urine was defrosted, centrifuged at 16,000*g* for 5 min, and diluted 1:4 with water containing 0.1% formic acid. Powdered tissues were extracted by using a protocol similar to that used for plasma samples but following repeated extractions. Briefly, from each tissue sample (~1 g), proteins were precipitated with 1500 μl of 2% formic acid in acetonitrile (the solvent volume for extraction was modified proportionately to the total weight of the samples). The samples were then vortexed vigorously for 5 min, placed in a sonicator bath for 10 min, and centrifuged (16,000*g* for 5 min). A second extraction was performed for each sample with 3 ml of acidified acetonitrile as described above. The two supernatants were pooled and dried under vacuum. Finally, the pellet was suspended in 100 μl of methanol:water:formic acid (50:50:0.1, v/v/v) and centrifuged at 16,000*g* for 5 min before analysis.

### UHPLC-MS/MS analysis

Plasma, urine, heart ventricles, liver, and pancreas samples were analysed according to Mele et al. [15] with minor modifications. Briefly, samples were run using an Accela UHPLC 1250 equipped with a linear ion trap-mass spectrometer (LTQ XL, Thermo Fisher Scientific) fitted with a heated-electrospray ionization probe (H-ESI-II). Separations were performed using a XSELECTED HSS T3 (50 × 2.1 mm), 2.5 μm particle size (Waters, Milford, MA, USA), with an injection volume of 5 μl, column oven temperature of 40 °C and elution flow rate of 0.3 ml/min. The initial gradient was 80 of 0.1% aqueous formic acid and 20% acetonitrile (in 0.1% formic acid), reaching 80% acetonitrile at 6 min. The MS conditions included: capillary temperature of 275 °C and source heater temperature of 250 °C, sheath gas flow of 60 units, auxiliary gas of 7 units, source voltage of 3.5 kV, and capillary voltage and tube lens of −39 and −88 V, respectively. Analyses were carried out using specific MS^2^ full scans corresponding to the putative phase II metabolites of UA and UB (glucuronide, sulphate, methyl, or a combination thereof), with collision induced dissociation (CID) equal to 35 (arbitrary units) (Table 1). After this first step, further specific MS^3^ analyses were carried out to unambiguously identify the compounds revealed in the first step. Pure helium gas was used for CID. Data processing was performed using Xcalibur software from Thermo Scientific. Quantification was performed with calibration curves of pure standards in the case of UA, UB, and UB-glucuronide (provided by Prof. O. Dangles, INRA, Avignon, France). UA-sulphate was expressed as UA equivalents, while UB-sulphate as UB equivalents.

**Table 1:** Urolithins detected in rat fluids and tissues

Urolithin	Test compounds	Rt (min)	[M−H]^−^ (*m/z*)	MS^2^ ions (*m/z*)	MS^3^ ions (*m/z*)^a^	Occurrence
Urolithin B	UB	3.62	211	167, 182		H, PC, L
	UB-sulphate	2.83	291	*211*	167	PM, U, H, PC, L
	UB-glucuronide	2.15	387	*211*, 175	167	PM
Urolithin A	UA	2.51	227	183, 159, 199		PM, H, PC, L
	UA-sulphate	1.79	307	*227*	198, 183	PM, H, PC, L

*PM* plasma, *U* urine, *H* heart, *PC* pancreas, *L* liver, *Rt* retention time

^a^MS^2^ ions in italic were those subjected to MS^3^ fragmentation for unambiguous identification

### Hemodynamic study

Invasive hemodynamic data were recorded in 39 rats (10 CTRL, 9 D3, 10 D3-UA, and 10 D3-UB). After anaesthesia with ketamine chloride (Imalgene, Merial, Milan, Italy; 40 mg/kg i.p.) plus medetomidine hydrochloride (Domitor, Pfizer Italia S.r.l., Latina, Italy; 0.15 mg/kg i.p.), the right carotid artery was cannulated with a microtip pressure transducer catheter (Millar SPC-320, Millar Instruments, Houston, TX, USA) connected to a recording system (Power Laboratory ML 845/4 channels, 2Biological Instruments, Besozzo, Italy) and systolic and diastolic blood pressures were determined. The catheter was then advanced into the left ventricle to measure the following parameters: left ventricular (LV) systolic pressure (LVSP), LV end-diastolic pressure (LVEDP), the maximum rate of ventricular pressure rise (+dP/dt_max_) and reduction (−dP/dt_max_), taken as indexes of myocardial mechanical efficiency, isovolumic contraction time (IVCT: duration of isovolumic contraction), and total cycle duration (Tcycle) (software package CHART B4.2).

### Isolation of adult left ventricular cardiomyocytes

From the heart of 6 CTRL, 6 D3, 5 D3-UA, and 4 D3-UB rats, individual LV myocytes were enzymatically isolated by collagenase perfusion. Briefly, the heart was rapidly excised through median sternotomy and mounted on a Langendorff isolated heart apparatus. The heart was then perfused at 37 °C by cannulating the aorta with the following sequence of solutions gassed with 100% oxygen: (1) calcium-free solution for 5 min to remove the blood, (2) low-calcium solution (0.1 mM) plus 1 mg/ml type 2 collagenase (Worthington Biochemical Corporation, Lakewood, NJ, USA) and 0.1 mg/ml type XIV protease (Sigma-Aldrich) for about 20 min, and (3) an enzyme-free, low-calcium solution for 5 min. Calcium-free solution contained the following (in mM, all chemicals from Sigma-Aldrich): 126 NaCl, 22 dextrose, 5.0 MgCl_2_, 4.4 KCl, 20 taurine, 5 creatine, 5 Na pyruvate, 1 NaH_2_PO_4_, and 24 HEPES (pH  =  7.4, adjusted with NaOH). The left ventricle was minced and shaken for 10 min. The cells were filtered through a nylon mesh and re-suspended in low-calcium solutions for 30 min (0.1 and 0.5 mM, for 15 min each). Then, cells were used for measuring sarcomere shortening and calcium transients.

### Cardiomyocyte contractility and Ca^2+^ transients

Mechanical properties of freshly isolated ventricular myocytes were assessed by using the IonOptix fluorescence and contractility systems (IonOptix, Milton, MA, USA). LV myocytes were placed in a chamber mounted on the stage of an inverted microscope (Nikon-Eclipse TE2000-U, Nikon Instruments, Florence, Italy) and superfused (1 ml/min at 37 °C) with a Tyrode solution containing (in mM; all chemicals from Sigma-Aldrich): 140 NaCl, 5.4 KCl, 1 MgCl_2_, 5 HEPES, 5.5 glucose, and 1 CaCl_2_ (pH 7.4, adjusted with NaOH). Only rod-shaped myocytes with clear edges and average sarcomere length ≥1.7 µm were selected for the analysis, using a 40× oil objective lens (NA: 1.3).

The cells were field stimulated at a frequency of 0.5 Hz by constant current pulses (2 ms in duration, and twice diastolic threshold in intensity; MyoPacer Field Stimulator, IonOptix). Load-free contraction of myocytes was measured with the IonOptix system, which captures sarcomere length dynamics via a Fast Fourier Transform algorithm. A total of 359 isolated ventricular myocytes were analysed (66 from CTRL hearts, 91 from D3, 100 from D3-UA, and 102 from D3-UB) to compute the following parameters: mean diastolic sarcomere length, fraction of shortening (FS), maximal rates of shortening (−dL/dt_max_) and re-lengthening (+dL/dt_max_), and time to 10, 50 and 90% of re-lengthening (time to basic length: TBL10%, TBL50%, and TBL90%). Steady-state contraction of myocytes was achieved before data recording by means of a 10 s conditioning stimulation. Sampling rate was set at 1 kHz.

In a subset of cells of each group (33 from CTRL hearts, 57 from D3, 63 from D3-UA, and 42 from D3-UB), Ca^2+^ transients were measured simultaneously with cell motion. Ca^2+^ transients were detected by epifluorescence after loading the myocytes with fluo-3-AM (10 µM; Invitrogen, Carlsbad, CA, USA) for 30 min. Excitation length was 480 nm, with emission collected at 535 nm. Fluo-3 signals were expressed as normalized fluorescence (f/f0: fold increase). The time course of the fluorescence signal decay was described by a single exponential equation, and the time constant (tau) was used as a measure of the rate of intracellular Ca^2+^ clearing [16].

### Electrophoretic and immunoblot assay

The hearts of 2 CTRL, 3 D3, 4 D3-UA, and 4 D3-UB were excised, and the left and right ventricles were weighed and immediately frozen at −80 °C, in order to determine the expression levels of the pro-inflammatory cytokine fractalkine (rabbit polyclonal ANTI-CX3CL1 antibody, 1:1000, Abcam, Cambridge, UK), and the following proteins involved in the intracellular calcium dynamics: the sarcoplasmic reticulum calcium ATPase 2 (SERCA2; rabbit polyclonal ANTI-SERCA2 ATPase antibody, 1:8000, Abcam), the regulatory protein phospholamban (PLB; mouse monoclonal ANTI-PHOSPHOLAMBAN antibody, 1:5000, Abcam) and the phosphorylated form of phospholamban (PLB-P; rabbit polyclonal ANTI- PHOSPHOLAMBAN (phospho S16) antibody, 1:1000, Abcam). The left and right ventricular tissue was mechanically fragmented in liquid nitrogen, and lysed with 300 µl of lysis buffer containing the following (all from Sigma-Aldrich): protease (1:100) and phosphatase (1:100) inhibitors, NaCl (150 mM), Tris–HCl (50 mM), EDTA (5 mM), Nonidet P-40 (1%), sodium fluoride solution (10 mM), sodium diphosphate dibasic (10 mM), SDS (0.1%) and sodium deoxycholate (0.5%). For each animal, equivalents of 50 µg of protein were separated on a 10% gradient SDS-PAGE gel for fractalkine and 4–20% gradient SDS-PAGE gel (Bio-Rad, Hercules, CA, USA) for SERCA2, PLB, and PLB-P. Membranes were blocked with 5% milk in Tris-Buffered Saline Tween-20 (TBS-T, Sigma-Aldrich) and incubated overnight at 4 °C with the primary antibody. After washing the membranes, a second incubation was performed for 1 h at RT with peroxidase-conjugated affinity purified goat anti-rabbit (goat Anti-Rabbit IgG Horseradish Peroxidase Conjugate, 1:8000; Bio-Rad, Hercules, CA, USA) or anti-mouse (goat Anti-Mouse IgG Horseradish Peroxidase Conjugate, 1:5000; Bio-Rad) secondary antibodies. Peroxidase activity was developed using the ECL Western blotting system (Amersham, Rahn AG, Zürich, Switzerland), according to the instructions of the manufacturer. Blots were scanned and the intensity of bands was quantified by means of the ImageJ software (NIH, Bethesda, MD, USA). All measurements were performed in technical triplicate. Actin (anti-actin rabbit polyclonal antibody, 1:5000, Sigma-Aldrich) was used as the loading control.

### Statistical analysis

The IBM SPSS statistical package (International Business Machines Corporation, Armonk, NY, USA, version 23) was used. Normal distribution of variables was checked by means of the Kolmogorov–Smirnov test. Data are reported as mean values ± SEM, except the urolithin concentrations in fluids and tissues which were not normally distributed and were thus expressed as median and interquartile range (IQR). Comparisons among groups involved two-way ANOVA followed by post hoc individual comparisons with the Bonferroni or Games-Howell test, when appropriate (hemodynamics), 2-factor Nested ANOVA for repeated measurements (cell mechanics, and Ca^2+^ transients), and non-parametric statistical test Kruskal–Wallis and U Mann–Whitney test (western blot data and urolithin concentrations). Differences were considered statistically significant at *p* < 0.05.

## Results

### Distribution and metabolism of urolithins

The administration of urolithin aglycones by i.p. injection guaranteed the presence of urolithin metabolites in circulation, as assessed by evaluating the plasma concentration of UA and UB and their putative phase II conjugates (Tables 1, 2). In the case of UB treatment (D3-UB), only UB metabolites were found: UB-sulphate was the predominant metabolite and was found in all plasma samples from rats treated with UB, while UB-glucuronide was present in some but not all samples (4/9). The profile of urolithin metabolites in circulation in the case of the animals treated with UA (D3-UA) was more complex: besides the aglycone form (UA) and UA-sulphate, some metabolites originating from the dehydroxylation of UA forming UB and further phase II conjugation were identified. In particular, both UB–glucuronide and UB-sulphate were detected in some D3-UA samples (Table 2). Similarly to what was reported for UB, UA-sulphate was the predominant urolithin metabolite in the circulation for D3-UA rats. A high inter-individual variability was observed in the plasma concentrations of urolithins for both D3-UB and D3-UA animals (Table 2). Control plasma samples from the CTRL and D3 groups did not contain detectable amounts of any urolithins. The urinary excretion of urolithin derivatives was very limited and only UB-sulphate was detected in D3-UB rats (Table 2).

**Table 2:** Concentrations (μM) of the urolithins detected in plasma and urine samples of rats treated with UB and UA

Metabolite	D3-UA			D3-UB			*p* value^b^
	Median (IQR)	Min–max	Samples^a^	Median (IQR)	Min–max	Samples	
Plasma							
Urolithin B-sulphate	0.000 (0.003)	0.000–0.012	1/6	0.339 (3.895)	0.065–5.858	9/9	§§§
Urolithin B-glucuronide	0.019 (0.033)	0.000–0.048	5/6	0.000 (0.032)	0.000–0.064	4/9	n.s.
Urolithin A	0.005 (0.007)	0.000–0.015	5/6	n.d.			–
Urolithin A-sulphate	0.220 (0.124)	0.121–0.394	6/6	n.d.			–
Urine							
Urolithin B-sulphate	n.d.			0.344 (5.622)	0.063–9.248	5/5	–

*n.d.* not detected (below LOD), *n.s* not significant

^a^Number of samples where each metabolite was quantified/detected

^b^§§§ indicates *p* < 0.001 significant differences between D3_UA and D3_UB (Mann–Whitney U test)

**Fig. 1:**
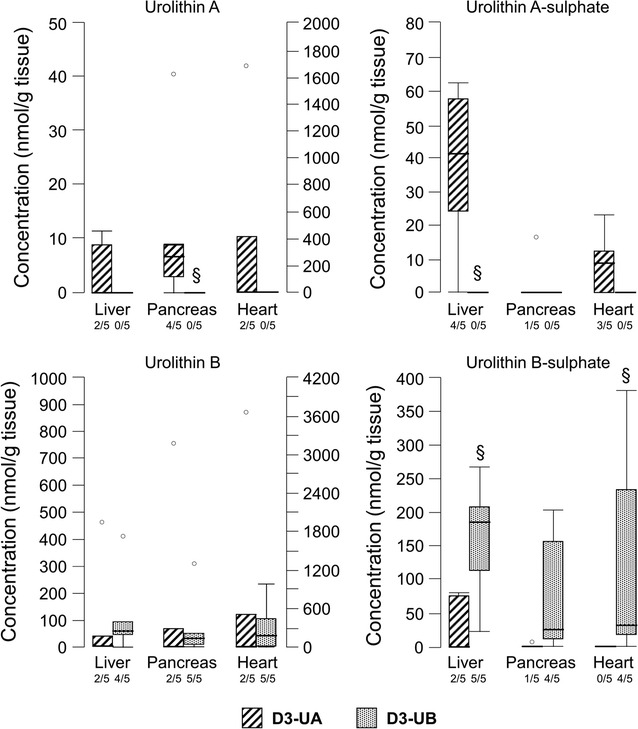
Accumulation of urolithin metabolites in tissues of rats treated with UA or UB (n = 5 for each group). Control samples (CTRL and D3 groups) did not contain detectable levels of these metabolites. The fraction under each column accounts for the number of samples where the compound was detected. For Urolithin A and Urolithin B, the y axes on the right refer to the concentration of the two metabolites in the heart tissue. ^§^ *p* < 0.05 significant differences between D3-UA and D3-UB (Mann–Whitney U test). °Outlier values

The analysis of tissue samples from the four experimental groups revealed the presence of urolithins in heart, pancreas, and liver of both D3-UB and D3-UA rats (Fig. 1), while these metabolites were absent in control samples. Aglycone and sulphate forms of urolithins, but not glucuronides, were detected in these tissues, although they were not detected in all the samples (Fig. 1), in line with the inter-individual variability highlighted for plasma and urine. Bioaccumulation of urolithin metabolites in the heart of D3-UB rats occurred in the form of UB and UB-sulphate (Fig. 1), without statistically significant differences between the concentrations of these two compounds. When D3 rats were treated with UA (D3-UA), UA, UA-sulphate and UB were found in the heart tissue. Again, no statistically significant differences were shown among metabolites for D3-UA heart samples. Urolithin A and B were also present in rat pancreatic and hepatic tissue, as both aglycone and sulphate. UB and UB-sulphate were detected in D3-UB pancreas and liver samples, while up to four metabolites (UA, UA-sulphate, UB, and UB-sulphate) were detected in D3-UA pancreas and livers. No statistical differences among metabolite concentrations for each treated group (D3-UB or D3-UA) and tissue (pancreas or liver) were observed, accounting for the absence of a prevailing compound. In general, UB, or its sulphated form, were found in tissues at higher concentrations with respect to UA or its sulphated form (Fig. 1).

### Blood glucose levels and body weight

All animals had normal blood glucose levels (105 ± 1 mg/dl), at the beginning of the experimental protocol. Two days after STZ injection, glycaemia significantly rose in diabetic rats compared with CTRL (410 ± 22 mg/dl vs 104 ± 1 mg/dl; *p* < 0.01). During the subsequent week, blood glucose levels increased slightly in untreated as well as urolithin-treated diabetic animals (~approximately 15%) and then remained stable until the end of the experimental protocol (after 3 weeks of hyperglycaemia: D3: 429 ± 18 mg/dl; D3-UA: 543 ± 22; D3-UB: 494 ± 28). A 7% decrease in body weight was observed during the first 2 weeks after STZ injection, in both untreated and treated diabetic animals. Subsequently, body mass exhibited only negligible changes in all experimental groups.

### Hemodynamics

**Fig. 2:**
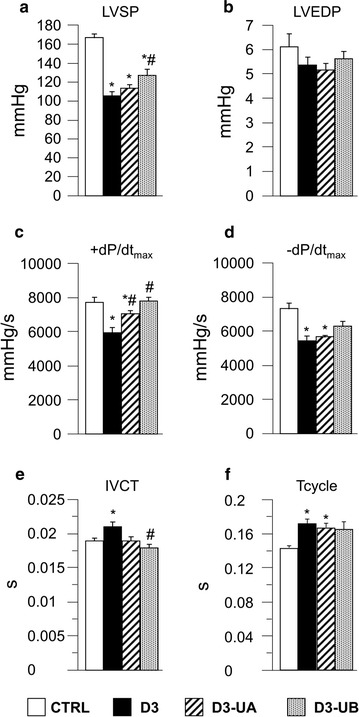
Hemodynamic measurements. Mean values ± SEM of left ventricular systolic pressure (LVSP; **a**), left ventricular end diastolic pressure (LVEDP; **b**), maximal rate of ventricular pressure rise (+dP/dt_max_; **c**), and decline (−dP/dt_max_; **d**), isovolumic contraction time (IVCT; **e**), and total cycle duration (Tcycle; **f**), measured in control rats (CTRL, n = 10), untreated diabetic rats (D3, n = 9), and urolithin A-treated (D3-UA, n = 10) or urolithin B-treated (D3-UB, n = 10) diabetic animals. *p < 0.05 significant differences vs CTRL; ^#^ *p* < 0.05 significant differences vs D3 (two-way ANOVA)

The heart rate measured under anaesthesia was similar in the four groups (193 ± 7 beats per minute, 205 ± 11, 204 ± 7, 209 ± 9 in CTRL, D3, D3-UA, and D3-UB, respectively). Compared with CTRL animals, D3 animals exhibited a global deterioration of hemodynamic performance, as indicated by the significant decrease in left ventricular systolic pressure (LVSP; Fig. 2a), the maximal rate of LV pressure rise (+dP/dt_max_) and decline (−dP/dt_max_) (Fig. 2c, d), and the marked prolongation of isovolumic contraction time (IVCT; Fig. 2e) and total cycle duration (Tcycle; Fig. 2f). The treatment with UB induced a significant recovery of most parameters, during both contraction and relaxation. Specifically, the values of LVSP, ± dP/dt_max_, IVCT and Tcycle approached those measured in CTRL animals (Fig. 2a, c–f). A lower effect was observed for UA, limited to the contraction phase (+dP/dt_max_ and IVCT; Fig. 2c, e). No significant differences among groups were found for LVEDP (Fig. 2b).

### Cell mechanics and calcium transients

**Fig. 3:**
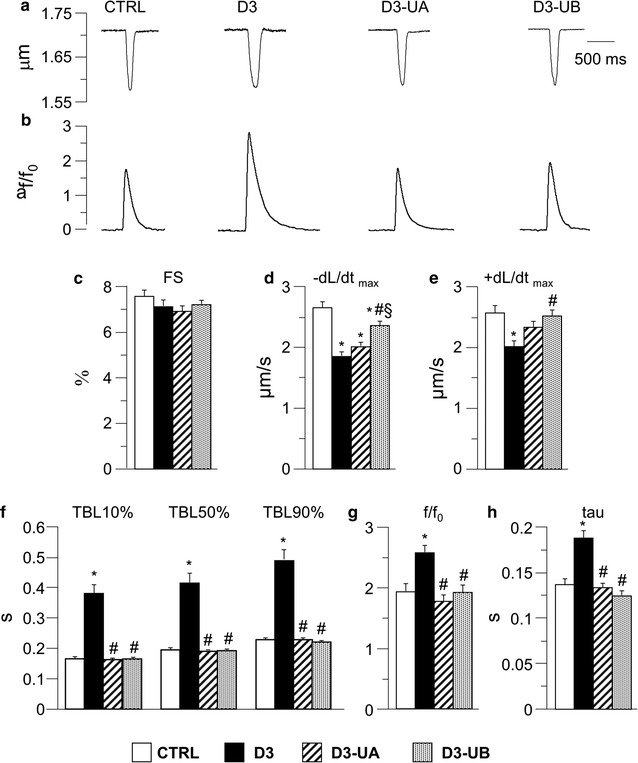
Cell mechanics and calcium transients. Representative examples of sarcomere shortening (**a**) and corresponding calcium transients (**b**; normalized traces: fold increase) recorded from CTRL, D3, D3-UA and D3-UB ventricular myocytes. In **c**–**h** *bar graphs* means values ± SEM of sarcomere fraction of shortening (FS; **c**), maximal rate of shortening (−dL/dt_max_; **d**), maximal rate of re-lengthening (+dL/dt_max_; **e**), time to 10, 50 and 90% of total cycle length (TBL10%, TBL50%, and TBL90%; **f**), calcium transient amplitude expressed as peak fluorescence normalized to baseline fluorescence (f/f0; **g**), and time constant of the intracellular calcium decay (tau; **h**), measured in CTRL (66 and 33 cells, for mechanics and calcium transients respectively), D3 (91 and 57 cells), D3-UA (100 and 63 cells), and D3-UB (102 and 42 cells). *p < 0.05 significant differences vs CTRL; ^#^ *p* < 0.05 significant differences vs D3; ^§^ *p* < 0.05 significant differences vs D3-UA (2-factor Nested ANOVA)

The average diastolic sarcomere length was comparable in all groups (1.732 ± 0.002 µm), as well as the fraction of shortening (Fig. 3c). Conversely, in accordance with the hemodynamic data recorded in vivo, contraction/relaxation properties and intracellular calcium dynamics were worsened in unloaded ventricular myocytes isolated from D3 hearts in comparison with CTRL (Fig. 3d–f). Specifically, D3 cardiomyocytes exhibited a significant decrease in the maximal rate of shortening (−dL/dt_max_; Fig. 3d) and re-lengthening (+dL/dt_max_; Fig. 3e), and a prolongation of re-lengthening times (TBL10%, TBL50%, and TBL90%; Fig. 3f). The impaired contractility in D3 cells was accompanied by a significant increase in calcium transient amplitude (+33%, Fig. 3g) and a prolonged tau (+37%, Fig. 3h).

Both urolithins led to an almost complete recovery of cellular mechanical properties and calcium dynamics, with only a slightly higher effect of UB (Fig. 3d–h).

### Electrophoretic and immunoblot assay

SERCA2, PLB, and PLB-P expression. Sarcoplasmic reticulum (SR) calcium reuptake rate is mainly regulated by the SERCA2 activity which, in turn, is modulated by a small molecular weight protein, the phospholamban (PLB). The phosphorylation status of PLB (PLB-P) is able to remove the inhibitory effect of PLB on SERCA2, resulting in increased SR-Ca^2+^ uptake and enhanced myocyte contractility and relaxation [17]. On the other hand, alterations in SERCA2 expression and/or activity are recognized among the major factors contributing to the ventricular dysfunction observed in diabetic cardiomyopathy [18–21].

**Fig. 4:**
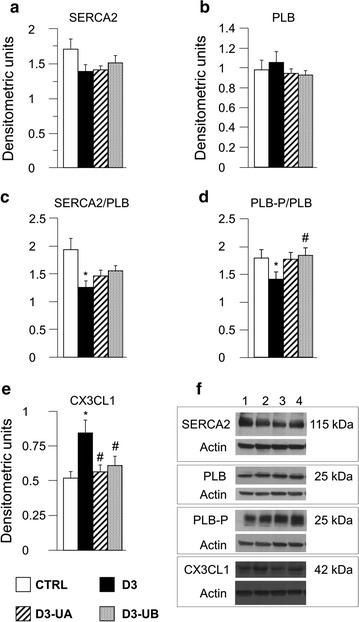
Expression levels of functional proteins and fractalkine by immunoblot assay. Average values ± SEM of SERCA2 (**a**), PLB (**b**), SERCA2/PLB ratio (**c**), PLB-P/PLB ratio (**d**), and fractalkine (**e**, CX3CL1) expression levels in left ventricular myocardium of CTRL (n = 2), D3 (n = 3), D3-UA (n = 4), and D3-UB (n = 4), in technical triplicate. Data are expressed in densitometric units. Sets of bands related to protein expression in 4 animals representative of the average behaviour observed in each group are reported in **f**. In *columns 1*–*4* CTRL, D3, DR-UA, and D3-UB animals. *p < 0.05 significant differences vs CTRL; ^#^ *p* < 0.05 significant differences vs D3 (non-parametric statistical test Kruskal–Wallis and U Mann–Whitney test)

A slight decrease (−19%; not significant) was observed in the expression level of SERCA2 associated with only negligible changes in PLB level, in D3 group as compared with CTRL (Fig. 4a, b), leading to a significant reduction in the SERCA2/PLB ratio (−35% vs CTRL, Fig. 4c). On the other hand, a reduced SERCA2/PLB ratio was shown to be associated with a parallel lessening of SERCA2 affinity for Ca^2+^, resulting in altered cardiomyocyte mechanics and depressed left ventricular function [22, 23]. Furthermore, diabetic hearts showed a pronounced decrease in PLB-P/PLB ratio (−21% vs CTRL, Fig. 4d) that, in the absence of substantial changes in the total PLB expression (Fig. 4b), suggested a reduction in the phosphorylated form of the protein. All these changes can account for the altered cellular contractile properties observed in diabetic cardiomyocytes. Although the effect of UB was slightly higher, both urolithins induced a marked amelioration of the two ratios whose values approached those measured in control hearts (Fig. 4c, d).

Fractalkine expression. Fractalkine, beside its action as a chemoattractant, is known to possess a detrimental effect on cardiac cells independently of inflammatory cell recruitment [24, 25]. The expression level of fractalkine (CX3CL1) was increased by hyperglycaemia (+48%; Fig. 4e) and significantly decreased by both urolithins (−33% for UA and −28% for UB; Fig. 4e).

## Discussion

Cardiomyopathy is a frequent and severe complication of both type-1 and type-2 diabetes [26], even in the presence of good glycaemic control. Cell oxidative stress and moderate inflammation constitute two closely related key factors in the pathogenesis of diabetic cardiomyopathy and play an important role in the initial stages of diabetes, leading to an altered tissue microenvironment associated with cell loss and/or dysfunction. This, in turn, triggers the progression of the disease towards the cardiomyopathy phenotype [1, 2, 27–29]. Thus, major attention should be focused on reducing the effects of the early key factors underlying the initial cellular biochemical and functional changes occurring in the diabetic heart.

In the last decade, different dietary relevant (poly)phenols have been tested as adjuvant therapeutic tools to counteract initial changes in the diabetic heart, possibly due to their antioxidant and anti-inflammatory properties [2, 8, 30, 31]. In this context, we recently showed that urolithins are able to reduce the pro-inflammatory response to high glucose levels in cultured neonatal cardiomyocytes and fibroblasts [13], suggesting that they can prevent the diabetes-induced microenvironmental changes triggering myocardial inflammation and functional impairment in the diabetic heart. These findings confirmed previous in vitro studies demonstrating the positive role of urolithin supplementation in different cell models, including anti-inflammatory activity in endothelial cells and macrophages [15, 32–36] and beneficial effects on lipid metabolism in adipocytes and hepatocytes [37]. However, the biological effects of in vivo urolithin administration in the setting of diabetes, as well as in other pathological conditions, remain to be characterized [38].

The present study demonstrates that in vivo urolithin treatment, besides reducing the inflammatory state of cardiac tissue, can prevent the occurrence of the early signs of cardiac dysfunction, as measured at organ and cellular-molecular levels, in a rat model of diabetes, after 3 weeks of hyperglycaemia.

In accordance with previous studies, showing that 3-weeks of diabetes corresponds to a transitional phase preceding overt DCM phenotype [2, 21, 39], we observed a deterioration of hemodynamic function and cardiomyocyte contractile properties, associated with altered intracellular calcium dynamics. In the absence of substantial variations in SERCA2 expression levels, abnormalities in calcium handling and contractility could be attributed to a decreased SERCA2 activity, mainly due to a reduced phosphorylation of PLB, as we actually observed. A depressed cell contractility, associated with an increased calcium transient and a prolonged *tau*, is consistently observed in this model of early diabetes [2]. A combination of several factors should likely be considered to interpret this behavior, including abnormalities in mitochondrial function, low pacing rate of cardiomyocytes, and SERCA2 activity. Mitochondrial dysfunction induced by cell oxidative stress was shown to reduce, in addition to ATP generation, the rate of calcium uptake into heart mitochondria [1]. The low pacing rate of cardiomyocytes (0.5 Hz) allows the removal of excess cytosolic calcium within the sarcoplasmic reticulum by SERCA2, independently of its reduced activity, and may explain the increased amplitude of the calcium transient following excitation. Nevertheless, the increase in calcium release does not result in an improvement in cell contractility due to the reduced ATP availability and the depressed myofilament calcium sensitivity, a hallmark of myofilament dysfunction in diabetic cardiomyopathy [40].

The functional impairment was coupled with initial tissue inflammation, as indicated by the significant increase in the expression levels of the pro-inflammatory cytokine fractalkine. This cytokine is expressed in early diabetes by cardiac fibroblasts and, to a lesser extent, by cardiomyocytes [2]. Fractalkine can act as either a soluble or membrane-bound mediator and signals through the G protein–coupled chemokine receptor CX3CR1, which is expressed on different cells, including cardiomyocytes [25, 41]. Accumulating evidence suggests that fractalkine, in addition to its role in chemotaxis, can directly affect the intracellular contractile machinery, leading to an altered cardiomyocyte mechanics [25]. Fractalkine has been shown to induce up-regulation of different phosphatases, including protein phosphatase 1 (PP1) and protein phosphatase 2A (PP2A), in failing myocardium [42]. These phosphatases are involved in dephosphorylation of proteins regulating the Ca^2+^ homeostasis in the myocardium, and thus essential regulators of myocardial function [43]. PP1 and PP2A dephosphorylate PLB, which is an intrinsic protein of the sarcoplasmic reticulum (SR) and a key regulator of SR-Ca^2+^-ATPase (SERCA2). SERCA2 activity is enhanced when PLB is phosphorylated, resulting in increased calcium re-uptake into the SR and enhanced contractility [17]. The fractalkine-induced reduction of the phosphorylated form of PLB, via up-regulation of phosphatases, can result in depressed SERCA2 activity and cardiomyocyte contractile dysfunction. We actually found a reduced PLB-P/PLB ratio associated with a significant impairment of intracellular Ca^2+^ dynamics and cardiomyocyte mechanics.

It is necessary to consider that fractalkine may also affect cardiomyocyte contractility by decreasing the phosphorylation of cardiac troponin I, as observed in control ventricular myocytes under isoproterenol stimulation [25]. PKA-induced phosphorylation of troponin I decreases myofilament calcium sensitivity, enhances sliding velocity, and contributes to the acceleration of relaxation [44]. Thus, a reduced phosphorylation of troponin I, by altering actin-myosin interactions, may have detrimental effects on cardiomyocyte mechanics.

The chronic administration of both urolithin A and B resulted in an almost complete recovery of cellular mechanical properties and intracellular calcium dynamics associated with a significant reduction in the tissue level of fractalkine. The beneficial effects of the two treatments could be ascribed primarily to the previously described action of urolithins in counteracting cellular oxidative stress and pro-inflammatory cytokine expression [13], which are intimately linked and constitute early detrimental changes in myocardial tissue of the diabetic heart, playing a pivotal role in DCM development.

The sulphate and glucuronide metabolites of UA and UB are the main metabolites appearing in circulation after consumption of ET-rich foodstuffs [45]. Direct i.p. administration of individual metabolites was the treatment of choice, as it allowed bypassing the microbial ellagitannin transformation step, that could have caused a substantial rise in inter-individual variability in urolithin production, allowing at the same time the discrimination of the putative effects exerted by UA and UB. The administration of aglyconic UA and UB did not limit the physiological relevance of these experiments since the aglycones were effectively transformed into phase II conjugates following i.p. injection. Sulphate conjugation favoured over glucuronidation in this model and UA-glucuronide, which has been previously described as the major urolithin metabolite in circulation after ET consumption [45–48], was not detected. This profile in phase II conjugates might be related to the by-pass of the intestinal barrier as absorption site, key in the glucuronidation of UA, and/or to the inhibition of UDP-glucuronyl transferases, considering the ability of UA to modulate phase II detoxifying enzymes [49]. On the other hand, the lack of substantial amounts of urolithin metabolites in rat urine was expected since the animals were starved prior to sacrifice, with urolithin metabolites having possibly already reached substantial clearance. The presence of metabolites in plasma several hours after treatment accounted for a high rate of enterohepatic recirculation of urolithins, a pharmacokinetic characteristic of these compounds [50]. Enterohepatic recirculation of i.p. administered urolithins is also supported by the dehydroxylation of UA forming UB, mediated by unknown urolithin-degrading bacteria in the intestinal lumen [51]. However, dehydroxylation of UA by a mammalian enzyme cannot be ruled out [52].

Urolithin metabolites accumulated substantially in rat tissues, including heart ventricles. The high capacity of the heart to accrue urolithins has recently been described [52], although to date no phase II metabolites have been reported. The high levels of UB metabolites in rat tissues is in line with a previous report highlighting the rapid accumulation of UB in rodent tissues compared to UA [52]. Interestingly, the higher concentration of UB and UB-sulphate in heart tissue in comparison with UA and UA-sulphate may explain the slightly higher efficacy of UB in preventing diabetes-induced myocardial dysfunction both at organ and cell levels in this work. Another point of note is that urolithins have been identified for the first time in pancreatic tissue. This observation opens new research scenarios in the framework of the study of the protective effects of urolithins in diabetes at the multiple organ level, especially considering the positive actions of some urolithins on insulin secretion [53].

Inter-individual variability in the production of urolithins is a well-known issue [12]. Although we hypothesised that inter-individual variability would have been drastically reduced by using i.p. administration, a high variation in metabolite concentrations was observed. Such variability can be attributed, at least in part, to animal-to-animal differences in metabolite clearance before sample collections. However, despite this variability in metabolite concentrations, the variability in measurements of cardiac function at organ and cellular-molecular levels was extremely low. This might account for a sustained and chronic effect of urolithins at the cardiac level, independent of their acute presence in circulation or even at heart tissue level.

## Limitations

Although we achieved the primary goal of our study, showing that in vivo urolithin administration succeeds in preventing early signs of cardiac dysfunction in diabetic rats, we acknowledge some limitations of this study. First, we used a rat model of type-1 diabetes. Nevertheless, it should be taken into account that cell oxidative stress and moderate myocardial inflammation constitute early detrimental changes which occur in both type-1 and type-2 diabetic hearts at the initial stages of the disease, playing a pivotal role in triggering the development of functional impairment. Secondly, we mainly attributed the cardiac functional recovery observed in urolithin treated diabetic rats to a reduced level of tissue inflammation, as suggested by the significant reduction of fractalkine levels in myocardial tissue of D3-UA and D3-UB hearts, but other potential mechanisms have not been explored and cannot be absolutely ruled out. Finally, the intracellular molecular mechanisms proposed to explain the effects of the pro-inflammatory cytokine fractalkine on cardiomyocyte mechanics and calcium dynamics are basically speculative and built on previously reported data.

## Conclusions

In conclusion, we showed here for the first time that in vivo urolithin administration may be able to prevent the initial inflammatory response of myocardial tissue to hyperglycaemia and the negative impact of the altered diabetic milieu on cardiac performance, as measured at cellular and organ levels. In line with previous data, our findings further support the idea that dietary (poly)phenols can constitute appropriate nutraceuticals and effective supplementary treatments for diabetes management and prevention of its complications.
